# Aberrant nonfibrotic parenchyma in idiopathic pulmonary fibrosis is correlated with decreased *β*‐catenin inhibition and increased Wnt5a/b interaction

**DOI:** 10.14814/phy2.12727

**Published:** 2016-03-20

**Authors:** Kristina Rydell‐Törmänen, Xiao‐Hong Zhou, Oskar Hallgren, Jonas Einarsson, Leif Eriksson, Annika Andersson‐Sjöland, Gunilla Westergren‐Thorsson

**Affiliations:** ^1^ Lung BiologyDepartment Experimental Medical ScienceLund UniversityLundSweden; ^2^ Department of BioSciencesRIA iMedAstraZeneca R&D GothenburgMölndalSweden; ^3^ Respiratory Medicine and AllergologyDepartment Clinical SciencesLund UniversityLundSweden; ^4^ Department Respiratory Medicine and AllergologySkåne University HospitalLundSweden

**Keywords:** ICAT, idiopathic pulmonary fibrosis, Wnt signaling, Wnt5a, *β*‐catenin

## Abstract

Idiopathic pulmonary fibrosis (IPF), an insidious disease with grave prognosis, is characterized by heterogeneous fibrosis with densely fibrotic areas surrounded by nonfibrotic normal‐looking tissue, believed to reflect a temporal development. The etiology is incompletely elucidated, but aberrant wound healing is believed to be involved. Embryonic signaling pathways, including Wnt signaling, are reactivated in wound healing, and we therefore aimed to investigate Wnt signaling, and hypothesized that Wnt signaling would correspond to degree of fibrosis. Material from 10 patients with IPF were included (four diagnostic biopsies and six donated lungs) and compared to healthy controls (*n* = 7). We investigated markers of Wnt signaling (*β*‐catenin, Wnt3a, ICAT, Wnt5a/b, DAAM1 and NLK) histologically in lung parenchyma with variable degree of fibrosis. Our results suggest that Wnt signaling is significantly altered (*P* < 0.05) already in normal‐looking parenchyma. The expression of Wnt3a and ICAT decreased (both *P* < 0.01) in IPF compared to healthy lungs, whereas *β*‐catenin, Wnt5a/b, DAAM1 and NLK increased (*P* < 0.05 for all). ICAT is further decreased in dense fibrosis compared to normal‐looking parenchyma in IPF (*P* < 0.001). On the basis of our results, we conclude that from a Wnt perspective, there is no normal parenchyma in IPF, and Wnt signaling corresponds to degree of fibrosis. In addition, *β*‐catenin and Wnt5a appears coupled, and decreased inhibition of *β*‐catenin may be involved. We suggest that the interaction between *β*‐catenin, ICAT, and Wnt5a/b may represent an important research area and potential target for therapeutic intervention.

## Introduction

Idiopathic pulmonary fibrosis (IPF) is an insidious disease, with grave prognosis and few treatment options (Gross and Hunninghake 2001). The etiology is incompletely elucidated, but it is generally believed that the fibrosis is the result of an aberrant wound healing (Kottmann et al. 2009). During wound healing and regeneration, embryonic signaling pathways are reactivated (Franco and Hess 2015), and one such pathway is Wnt signaling, which has previously been implicated in the pathogenesis of IPF (Chilosi et al. 2003; Konigshoff et al. 2008). Wnt is a group of 19 ligands that transduce cell–cell interaction and are involved in, for example, migration, differentiation, and proliferation (Baarsma et al. 2013). Wnt signaling is commonly divided into canonical (*β*‐catenin mediated) and noncanonical (non‐*β*‐catenin mediated) signaling, where the noncanonical signaling can be further divided into two pathways; Planar Cell Polarity (Wnt/PCP) pathway and the Ca^2+^‐dependent pathway (Wnt/Ca^2+^). In this study, we investigated *β*‐catenin‐mediated signaling as well as Wnt/PCP and Wnt/Ca^2+^ in IPF.

IPF is characterized by heterogeneous fibrosis, with areas of dense fibrosis adjacent to normal‐looking tissue with intact alveolar structures, and it is generally believed that this pattern represents a temporal development, where densely fibrotic areas represent advanced disease (Gross and Hunninghake 2001). A previous study, investigating Wnt signaling over time in a mouse model of pulmonary fibrosis, suggested that Wnt signaling appeared to be related to the degree of fibrosis/remodeling (Andersson‐Sjöland et al. 2016). The aim of this study was therefore to map and investigate markers of Wnt signaling in tissue areas with variable fibrosis severity, presumably reflecting disease duration. We hypothesized that Wnt signaling is distorted in IPF, and the alterations corresponds to degree of fibrosis, that is, the more fibrosis the more alterations.

Our results suggest that the normal‐looking parenchyma in IPF is not truly normal, but appears to be highly involved in progression of fibrosis, and Wnt signaling varies depending on degree of fibrosis. Furthermore, Wnt5a/b and *β*‐catenin appears to be involved in an intimate relationship, and we identified decreased expression of the *β*‐catenin inhibitor ICAT to correspond to the degree of fibrosis.

## Methods

### Patient material and controls

Fibrotic lung material was obtained from explanted lungs (*n* = 6) and diagnostic open chest biopsies (*n* = 4). Control material (*n* = 7) was obtained from explanted lungs from healthy individuals fulfilling the criteria for lung donation after brain stem death but unmatched to a recipient. All protocols were reviewed and approved by the local ethic committee. Patient characteristics are further described in Table 1.

**Table 1:** Patients characteristics

	IPF (*n* = 10)	Healthy controls (*n* = 7)
Age (years)		
31–45	1	2
46–65	4	5
66–85	5	0
Sex (m/f)	5/5	4/3
Smoking history^a^		
Nonsmoker	2	5
Ex smoker	8	1
Disease duration^b^ (years)		
<2	2	
>2	4	
FVC (l)		
Newly diagnosed IPF	2.9 ± 0.4	
Late‐stage IPF	1.8 ± 0.9	
FVC (%)		
Newly diagnosed IPF	64.5 ± 7.3	
Late‐stage IPF	58 ± 29.7	
FEV1 (l)		
Newly diagnosed IPF	2.4 ± 0.3	
Late‐stage IPF	1.5 ± 0.7	
FEV1 (%)		
Newly diagnosed IPF	86.0 ± 4.1	
Late‐stage IPF	55.7 ± 21.2	
DLCO (%)		
Newly diagnosed IPF	50.5 ± 7.3	
Late‐stage IPF	29.2 ± 4.4	

a Unknown in one healthy control.

b 4 IPF patients, where biopsies were taken for diagnostic purposes is not included; these patients were diagnosed in 2012–13 and still alive in April 2015.

### Immunohistochemistry (IHC)

Paraffin‐embedded tissue was section into 4‐*μ*m‐thick sections, and stained. Briefly, sections were rehydrated and equilibrated in TBS before antigen retrieval, either by heat in low pH buffer, or enzymatically with Proteinase K (20 *μ*g/ml, 37°C/30 min), before primary antibody was applied (omitted for controls) and incubated 1.5 h (RT). Slides were rinsed and secondary antibody, visualized using DAB, counterstained in HTX and mounted. The following antibodies were used to visualize markers of Wnt signaling: Wnt3a (1:300, 09‐162, EMD Millipore Corp. Billerica, MA), Wnt5a/b (1:100, ab72583, Abcam, Cambridge, UK), *β*‐catenin (1:100, AF1329, R&D Systems, Minneapolis, MN), Disheveled‐associated activator of morphogenesis 1/DAAM1 (1:200, ab71327, Abcam), Inhibitor of *β*‐catenin and TCF‐4/ICAT (1:50, ab197916, Abcam) and Nemo‐like kinase/NLK (1:100, ab26050, Abcam). For double stainings to visualize cell types, the following antibodies were used in combination with *β*‐catenin; Prosurfactant Protein C (ProSpC, 1:1000, ab90716, Abcam), pan‐cytokeratin (1:100, ab27988, Abcam) and Vimentin (1:500, ab92547, Abcam), as well as ICAT and NLK. Fluorescent secondary antibodies were used (dilutions and pretreatments were identical when applicable).

### Histological analysis

**Figure 1:**
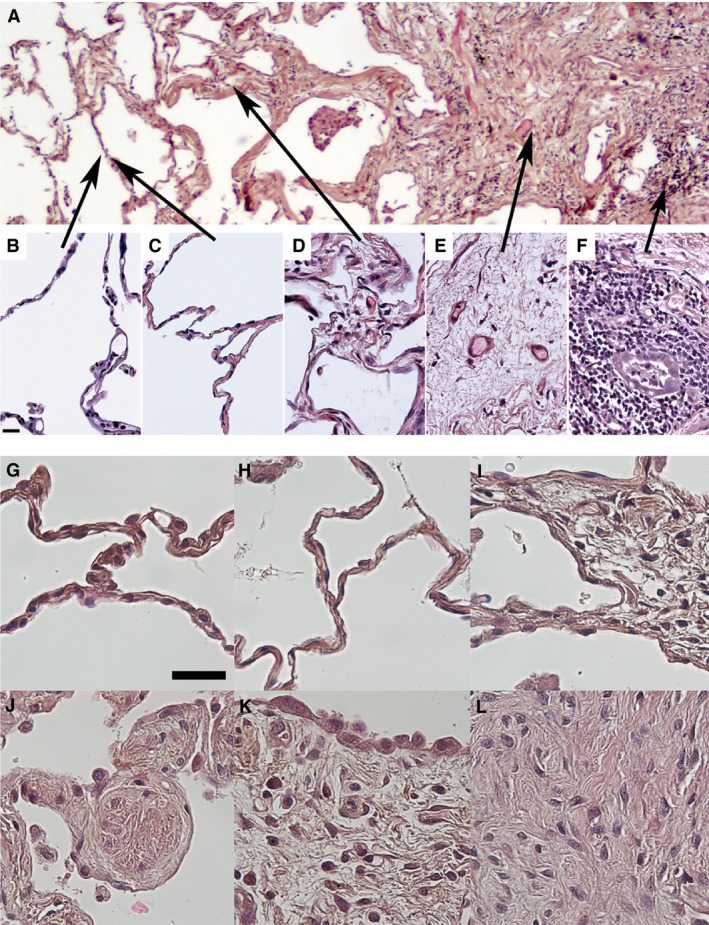
Illustrative images of the different compartments described within this study and enlargements of specific structures (A) *Healthy:* Healthy parenchyma from control subjects (B), *Normal:* Normal‐looking parenchyma from IPF patients was not found in all IPF samples (C), *Border:* Border zone adjacent to densely fibrotic areas, or between densely fibrotic and normal‐looking tissue (D), *Dense:* Densely fibrotic parenchyma, few structures can be identified beside scattered airways and vessels (E), *Inflammation*: Inflammatory zone with infiltrating mononuclear cells (F). High magnification images of specific structures; The healthy lung parenchyma (G) and normal‐looking lung parenchyma from an IPF patient (H) are ultrastructurally very similar. In border zones, the alveolar septa were thickened (I) and small nodular foci were found (J). Both in border zones (J) and overlying densely fibrotic areas (K) altered alveolar epithelium was found. In densely fibrotic areas, no alveolar structures were found (K, L). Scale bar in B represents 20 *μ*m and is applicable for images B–F, and scale bar in G represents 30 *μ*m and is applicable for images G–L.

Analyses were performed using digital imaging and ImageJ (v1.44j; Wayne Rasband, NIH). The number of positively labeled cells/nuclei were counted and related to the tissue area (mm^2^). For each marker, 6–20 images were taken of each lung section, the larger number in IPF lungs, where several different tissue types were found. Regions were defined based on histologic appearance (Fig. 1A). To our knowledge no accepted method for this has been published, and we therefore defined them as follows:

*Healthy:* Healthy parenchyma from control subjects (*n* = 6–7 for all markers, Fig. 1B)
*Normal:* Normal‐looking parenchyma from IPF patients, found in all diagnostic biopsies and 1–2 of the explanted lungs (*n* = 4–6 depending on marker, Fig. 1C).
*Border:* Adjacent to densely fibrotic tissue, and commonly between densely fibrotic and normal tissue (when present) (Fig. 1D), characterized by thickening of the alveolar walls and often altered epithelium.
*Dense:* Densely fibrotic parenchyma, few structures can be identified beside scattered airways and vessels. Found in all IPF lungs (*n* = 10 for all markers (Fig. 1E)).
*Inflammation:* Inflammatory zone with infiltrating mononuclear cells (Fig. 1F).

### Statistical analysis

Differences between groups were tested using Kruskal–Wallis combined with LSD using Analyse‐it for Microsoft Excel (Analyse‐it Software, Ltd, Leeds UK). *P* < 0.05 was considered significant, and data are given as mean ± SD.

## Results

### Histological characterization

Normal‐looking tissue was found in all diagnostic biopsies (Fig. 1G–H), but not in all explanted lungs. Border zones (Fig. 1I) with small fibrotic nodules (Fig. 1J) were found in all IPF patients. Densely fibrotic areas, present in all IPF lungs, were surrounded by abnormal epithelium (Fig. 1K), and characterized by fibrotic tissue without alveolar structures (Fig. 1L). In inflammatory areas, a massive variation was present, likely due to individual inflammatory processes.

**Figure 2:**
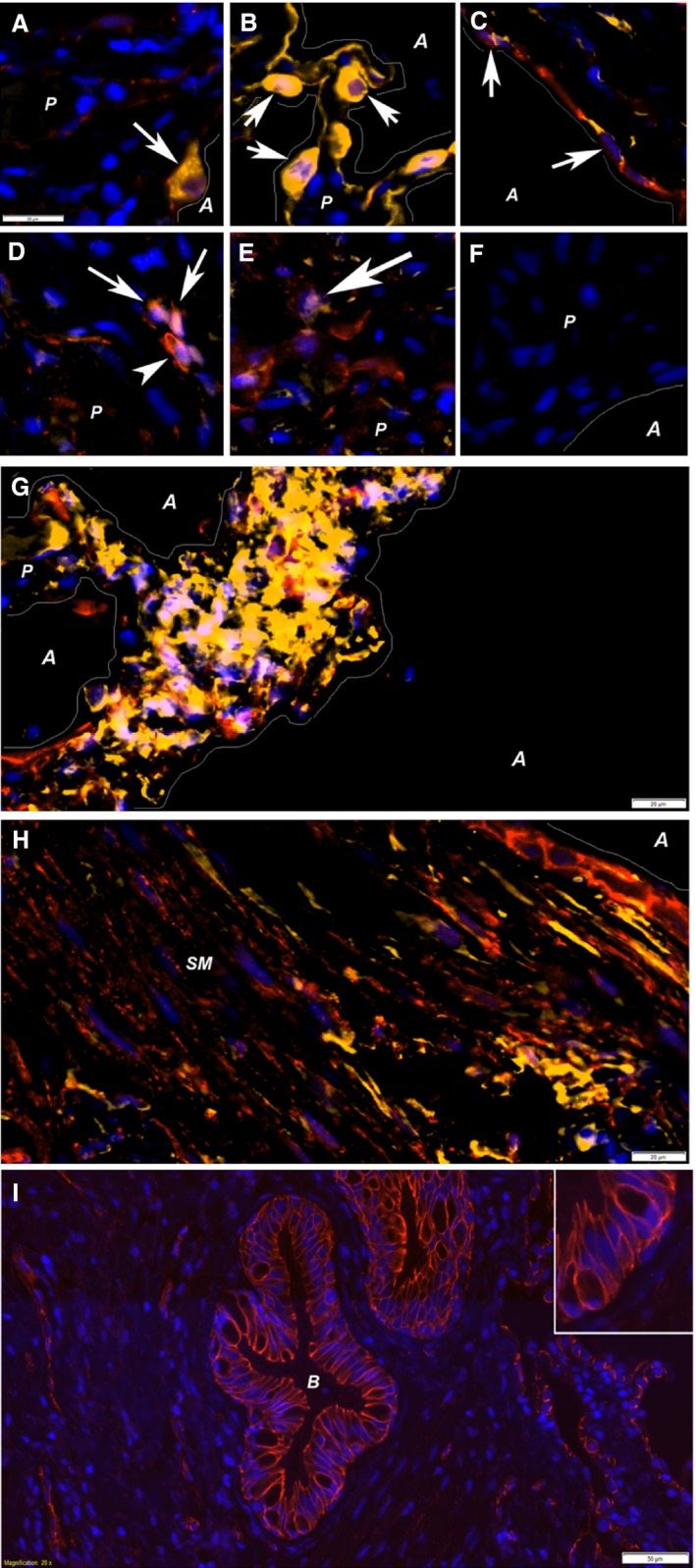
Double stainings with β‐catenin. Several double stainings with *β*‐catenin was performed to investigate the cell types activated. These double stainings showed colocalization (arrows) of *β*‐catenin (red staining in all images) with Prosurfactant Protein C (A), pan‐cytokeratin (B), vimentin (C), NLK (D), and ICAT (E). These antibodies were all visualized with Cy3 (yellow labeling), and DAPI (blue) was used to visualize nuclei. Negative controls, omitting the secondary antibody (F) showed no positive labeling. *β*‐catenin/vimentin‐positive cells (purple nucleus in yellow cells) were also found in foci within border zones (G) as well as within smooth muscle bundles (H) in association with bronchial epithelium. Vimentin‐positive epithelial cells (C) suggest presence of EMT. Interestingly, *β*‐catenin was found both in cellular junctions/in the cell membrane (D, arrowhead and I) and within nuclei (arrows in A, B, C, D and E). All images, except D, E, and I are taken in border zones, whereas D and E are taken in densely fibrotic tissue where no alveolar structures were found and I shows a bronchiole. Structural elements within the images are marked; lines illustrate the border between parenchymal (*P*) tissue and alveolar (*A*) spaces, in addition to smooth muscle bundles (*SM*) and bronchioles (*B*). Scale bars represent 20 *μ*m in A–H, 50 *μ*m in I, and scale bar in A is applicable to images A–F.

Double labeling shows *β*‐catenin copositivity with ProSpC (Fig. 2A), pan‐cytokeratin (Fig. 2B), Vimentin (Fig. 2C,G and H), NLK (Fig. 2D) and ICAT (Fig. 2F). Vimentin was found in both epithelial cells (Fig. 2C) as well as in clusters within tissue of border zones (Fig. 2G) and in smooth muscle bundles (Fig. 2H). In airway epithelium *β*‐catenin labeling displayed a clear junctional pattern, consistent with the presence of *β*‐catenin bound to cadherins in junctional complexes (Fig. 2I).

### Decreased Wnt/3a expression in IPF

**Figure 3:**
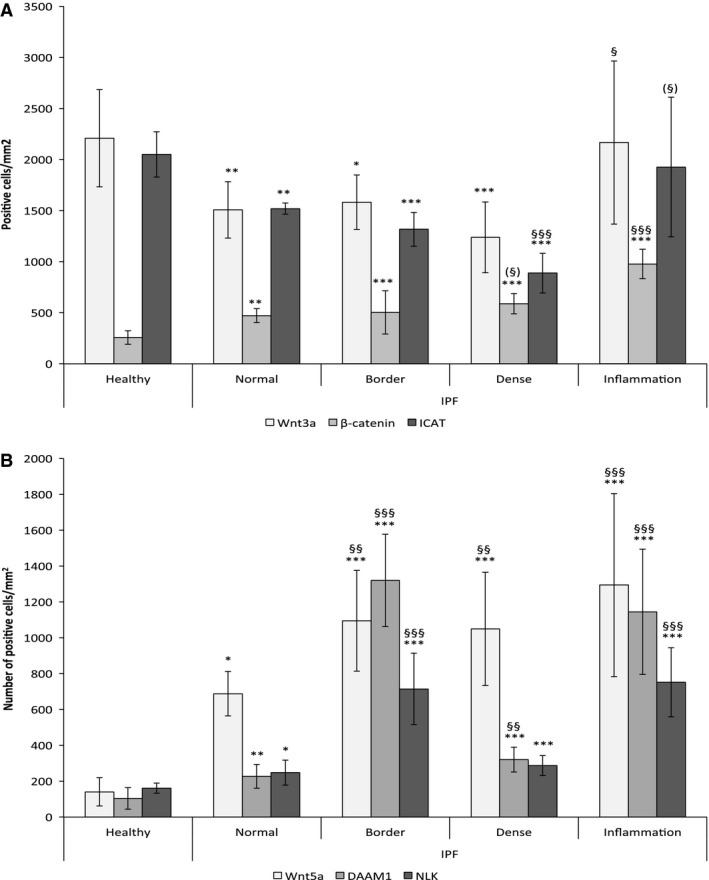
Expression of β‐catenin, ICAT, Wnt3a, DAAM1, NLK and Wnt5a, and representative illustrations of tissue labeling. Expression of Wnt3a, β‐catenin, and ICAT in healthy lungs, and in different regions of fibrotic lung (A). In densely fibrotic and border zones *n* =10 and in healthy lungs *n* = 6 for all parameters. Normal‐looking parenchyma *n* = 6 (*β* ‐catenin, ICAT) or 7 (Wnt3a); Inflammation n = 8 (*β* ‐catenin), *n* = 9 (Wnt3a), and *n* = 10 (ICAT). Expression of Wnt5a, DAAM1, NLK and in healthy lungs, as well as in different regions of fibrotic lung (B). For densely fibrotic areas *n* = 10 and in healthy lungs *n* = 6 for all parameters. Normal‐looking parenchyma *n* = 8 (DAAM1), *n* = 6 (NLK, Wnt5a); Border zone *n* = 6 (DAAM1, Wnt5a), *n* = 7 (NLK, Wnt5a); *n* = 6 (NLK); Inflammation *n* = 8 (DAAM1, Wnt5a), *n* = 7 (NLK). The variation in inflammatory areas is massive, likely due to very individual inflammatory pattern. *Denotes difference compared to healthy lungs, and § denotes difference compared to normal‐looking areas of IPF lungs. (§) Marks strong tendency *P *= 0.05–0.07.

**Figure 4:**
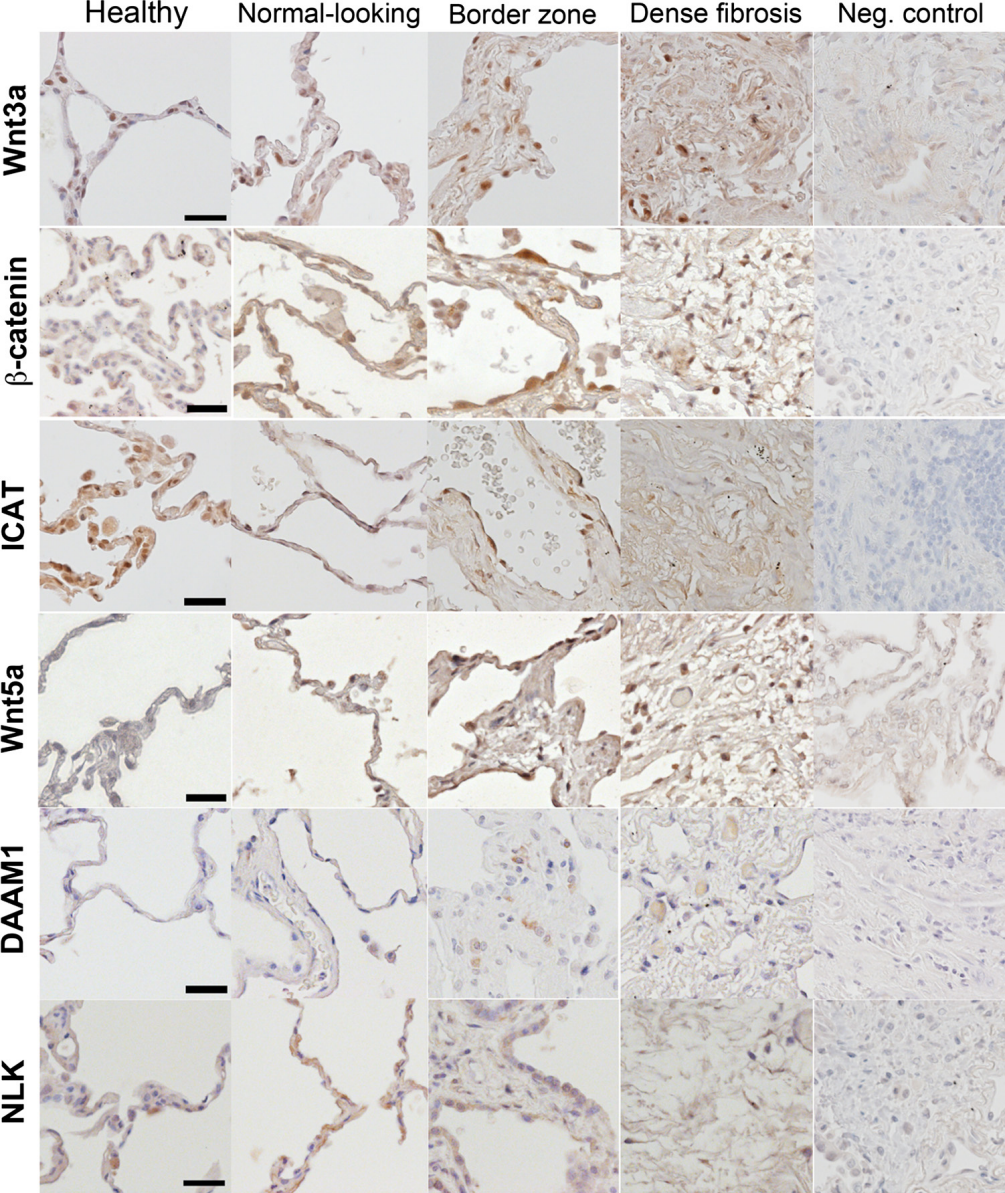
Illustrative images of tissue distribution. Tissue distribution of the investigated mediators; Wnt3a, *β*‐catenin, ICAT, Wnt5a/b, DAAM1 and NLK, in all healthy lungs as well as in the different compartments of IPF lungs. In negative controls, the primary antibody was omitted, and all visualization was done with DAB. Scale bars represent 30 *μ*m and are applicable to all images.

Our results show that Wnt3a‐positive cells is significantly decreased (25–33%) in IPF compared to controls (Figs. 3A, 4). No difference was found within lungs from IPF patients.

Both epithelial and endothelial cells were Wnt3a‐positive, as were spindle‐shaped mesenchymal cells likely to be fibroblasts/myofibroblasts.

### Increased β‐catenin signaling in IPF

The number of *β*‐catenin‐positive nuclei (Figs. 3A, 4) was significantly increased (*P* < 0.01) in normal‐looking parenchyma in IPF (472 ± 68 nuclei/mm^2^), as well as in border zones (503 ± 211), dense fibrosis (589 ± 99), and inflammatory zones (929 ± 130) compared to healthy controls (257 ± 66). A very strong tendency was seen toward increased nuclear localization in dense fibrosis compared to normal‐looking parenchyma (*P* = 0.059) in IPF.

Most labeled cells were found to be epithelial or endothelial cells, but also mesenchymal cells, likely fibroblasts/myofibroblasts, were positive.

### Decreased inhibition of β‐catenin by “Inhibitor of β‐catenin and TCF‐4” (ICAT)

Expression of ICAT (Figs. 3A, 4) was significantly decreased throughout the fibrotic lung (p < 0.01), compared to healthy lungs. In addition, the expression in dense fibrosis (888 ± 195 cells/mm^2^) is significantly decreased (*P* < 0.001), compared to normal‐looking parenchyma (1520 ± 54).

Importantly, ICAT‐positive cells appear to be the same types as *β*‐catenin‐positive cells.

### Increased noncanonical signaling in IPF

#### Wnt5a/b

Wnt5a is increased in fibroblasts isolated from IPF patients (Vuga et al. 2009). Wnt5b shares 84% homology with Wnt5a, and is functionally very similar. Our results suggest that the number of Wnt5a/b‐positive cells is significantly (*P* < 0.05) increased five to ninefold in the fibrotic lung (Figs. 3B, 4). Furthermore, the number of positive cells was significantly increased in border zones (1095 ± 281 cells/mm^2^) and dense fibrosis (1050 ± 316) compared to normal‐looking parenchyma (688 ± 124).

#### Disheveled‐associated activator of morphogenesis 1 (DAAM1)

To investigate Wnt/PCP signaling we studied expression and localization of DAAM1 (Figs. 3B, 4), which was significantly increased (*P* < 0.01) in normal‐looking parenchyma (227 ± 66 cells/mm^2^), border zones (1320 ± 257), and dense fibrosis (320 ± 69) in IPF patients compared to healthy donors (104 ± 60 cells/mm^2^). DAAM1 also displayed a 1.5 to 6‐fold increased in border zones, dense fibrosis and inflammatory zones, compared to normal‐looking parenchyma in IPF (*P* < 0.01).

#### Nemo‐like kinase (NLK)

Nemo‐like kinase is activated by the Wnt/Ca^2+^ signaling pathway, and inhibits *β*‐catenin activation. Results (Figs. 3B, 4) show significantly increased NLK expression in normal‐looking (248 ± 70 cells/mm^2^), border (715 ± 199), and densely fibrotic (287 ± 56) tissue from IPF patients expressed compared to healthy controls (161 ± 28 cells/mm^2^). In addition, NLK expression was also increased (3 times) in both border zones and inflammatory zones, compared to normal‐looking parenchyma.

## Discussion

To our knowledge, this study presents the first histological mapping different markers of Wnt signaling in IPF, providing a unique characterization areas with different tissue density that likely represents different temporal phases of IPF. We conclude that, the normal looking, nonfibrotic parenchyma is significantly affected, with altered Wnt signaling and increased *β*‐catenin activation and decreased inhibition.

The overall aim was to perform a histologic investigation of Wnt signaling in IPF, which we hypothesized would correspond to degree of fibrosis; no changes in normal‐looking parenchyma and severe alterations in fibrotic areas. The histologic approach allowed a more detailed quantification and importantly, this dimension is often overlooked as analyses of fibrotic lung often rely on tissue homogenates. The spatial pattern of fibrosis has been suggested to represent a temporal development (Gross and Hunninghake 2001), and this approach can therefore be interpreted as mapping Wnt signaling longitudinally within the same lung.

*β*‐catenin has been recognized as an important participant in the development of fibrosis, since the first description by Chilosi et al. who showed *β*‐catenin to be increasingly active in IPF and suggested aberrant activation to be a key event in IPF (Chilosi et al. 2003). The involvement of *β*‐catenin has since then been extensively described and investigated (Konigshoff et al. 2008; Enzo et al. 2015). However, most studies focus on the activation/stabilization of *β*‐catenin. In contrast, we chose to investigate one of the inhibitors, ICAT, which dissociates the interaction between *β*‐catenin and different transcription complexes (Daniels and Weis 2002). Interestingly, we found ICAT to be decreased throughout the lung, and the combination with increased *β*‐catenin, may suggest a dysregulated control of *β*‐catenin signaling. Decreased expression of Wnt3a [in accordance with previous studies (Konigshoff et al. 2008)], suggests that the nuclear relocalization of *β*‐catenin was not initiated by canonical signaling. One alternative mechanism is TGF‐*β*‐induced stabilization (Gosens et al. 2010; Kumawat et al. 2014), which is known to result in increased Wnt5a expression and subsequent ECM synthesis (Kumawat et al. 2013). Intriguingly, a study by Zhou and colleagues showed an interaction between *β*‐catenin and TGF‐*β* signaling pathways, with a direct interaction between Smad3 and *β*‐catenin, and this interaction was abrogated by overexpression of the *β*‐catenin inhibitor, ICAT (Zhou et al. 2012). Furthermore, Wnt5a has also been shown to induce stabilization of *β*‐catenin (Mikels and Nusse 2006). Our data, in combination with previous studies thus suggest an intriguing affair between *β*‐catenin, TGF‐*β,* and Wnt5, with a potential key role for ICAT that needs to be further investigated in vivo as well as in vitro.

In contrast to our expectations, our results suggest that the normal looking, nonfibrotic parenchyma is not normal but severely altered. Since the tissue looked morphologically normal, the alteration described in our study likely represents some of the earliest alterations in IPF. Based on this, it can be suggested that the normal‐looking parenchyma represent the actual battlefront in the development of fibrosis, and should therefore be the focus when investigating disease initiation and early development.

Importantly, some Wnt ligands share a very high degree of homology (especially Wnt3/3a and Wnt5a/b) the specificity of the antibodies can be questioned. On the basis of the peptide sequence used to generate the Wnt3 and Wnt5 antibodies, we cannot exclude some promiscuous binding. Importantly, the homology between Wnt3/3a and Wnt5a/b is relatively low, thus cross‐labeling is unlikely. Furthermore, results from an MS study (data not shown) failed to detect Wnt3 in healthy or fibrotic lung (suggesting that the labeling is truly Wnt3a, and not a combination with Wnt3), whereas both Wnt5a and Wnt5b were detected. Both Wnt5a and Wnt5b have been found to be upregulated in parallel in response to TGF‐*β* (Kumawat et al. 2013), and it is thus likely that the histologically detected increase is composed of both Wnt5a and Wnt5b. Unfortunately, elucidating of the relative expression of Wnt5a and 5b is beyond the scope of this study, but our results identify this as an area of interest for further studies.

DAAM1 facilitates Wnt‐induced Dvl‐Rho complex formation, which is essential for Wnt/PCP and necessary for regeneration of epithelial and endothelial cell layers (Cirone et al. 2008; Andreeva et al. 2014). NLK functions as a proline‐directed kinase, and exert its controlling effects on *β*‐catenin signaling by phosphorylating LEF1 and rendering it inactive (Ishitani and Ishitani 2013). Neither DAAM1 nor NLK has been previously investigated in IPF, but based on their respective functions and known effects, both may contribute to the development of fibrosis.

A more through histologic approach, using double and triple stainings, allowed us to determine which cells that were involved. We could determine that *β*‐catenin was often activated in epithelial cells (both type I and II), which rarely expressed the *β*‐catenin inhibitors, ICAT and NLK. These inhibitors were more commonly found in vimentin‐positive cells within the tissue, likely fibroblasts and/or myofibroblasts. This suggests and intriguing pattern, with activated *β*‐catenin in epithelial cells lacking inhibitory signals. Accordingly, the lack of *β*‐catenin inhibitors in epithelial cells clearly warrants further investigations. Interestingly, very few cells were positive for epithelial markers (pan‐cytokeratin and prosurfactant protein C) in the densely fibrotic areas, whereas vimentin‐positive cells were commonly found in these areas, as well as in clusters beneath the epithelium and in smooth muscle bundles. *β*‐catenin‐positive cells were commonly found within these areas of vimentin‐positive cells. Within healthy epithelium, *β*‐catenin‐positive labeling was found in adherens junctions as previously described (Salomon et al. 1997; Chilosi et al. 2003).

For ethical reasons, it is more or less impossible to study IPF longitudinally, but our results suggest that the degree of fibrosis represent a temporal development. It was clear that explanted lungs were more fibrotic than diagnostic biopsies, as normal‐looking parenchyma was found in all biopsies, but only some of the explanted lungs. Furthermore, the individual pharmacological treatments were inadequately known for the patients, and we believe the great variability in the inflammatory zones, were due to variable therapeutic treatments. Within these areas labeled cells were most often located adjacent to or interspaced between the immune cells, resembling lymphoid aggregates, previously described (Todd et al. 2013).

Idiopathic pulmonary fibrosis is mainly known as a disease of 60+ males, yet our sample cohort included 50% females and 50% were under 65 years of age. All patients had been diagnosed with IPF following standard clinical criteria. The control material originates from somewhat younger individuals (aged 31–65).

On the basis of our results, we conclude that the normal looking, nonfibrotic parenchyma may represent the fibrotic battlefront and suggest the interaction between *β*‐catenin, ICAT and Wnt5a/b as an important research area and potential target for therapeutic intervention.

## Conflict of Interest

None declared.
